# *Arabidopsis halleri*: a perennial model system for studying population differentiation and local adaptation

**DOI:** 10.1093/aobpla/plz076

**Published:** 2019-11-27

**Authors:** Mie N Honjo, Hiroshi Kudoh

**Affiliations:** Center for Ecological Research, Kyoto University, Hirano, Otsu, Shiga, Japan

**Keywords:** Altitudinal adaptation, clonal propagation, common garden experiment, genome scan, life history, metalliferous habitat, perennial, RNA-Seq

## Abstract

Local adaptation is assumed to occur when populations differ in a phenotypic trait or a set of traits, and such variation has a genetic basis. Here, we introduce *Arabidopsis halleri* and its life history as a perennial model system to study population differentiation and local adaptation. Studies on altitudinal adaptation have been conducted in two regions: Mt. Ibuki in Japan and the European Alps. Several studies have demonstrated altitudinal adaptation in ultraviolet-B (UV-B) tolerance, leaf water repellency against spring frost and anti-herbivore defences. Studies on population differentiation in *A. halleri* have also focused on metal hyperaccumulation and tolerance to heavy metal contamination. In these study systems, genome scans to identify candidate genes under selection have been applied. Lastly, we briefly discuss how RNA-Seq can broaden phenotypic space and serve as a link to underlying mechanisms. In conclusion, *A. halleri* provides us with opportunities to study population differentiation and local adaptation, and relate these to the genetic systems underlying target functional traits.

## Teaser

One of the most fascinating observations on plants in natural habitats is the spatial co-variation between functional traits and natural environments. Here, we introduce a small perennial clonal herb, *Arabidopsis halleri* and its life history as a model system to study local adaptation along environmental gradients. Many studies using this species have addressed altitudinal adaptation in diverse functional traits and population differentiation in response to heavy metal contamination in soils. The species provides us with opportunities to study population differentiation and local adaptation, and relate these to the genetic systems underlying target functional traits using recent molecular tools.

## Introduction

One of the most fascinating observations on plants in natural habitats is the spatial co-variation between functional traits and natural environments. In particular, when the phenotypic difference has a genetic basis, we expect that the spatial variation in selection might have shaped the co-variation between phenotype and environment. Population differentiation refers to genetic differentiation between populations in a trait or a set of traits (Linhart and Grant 1996). Most ecological traits show a certain level of phenotypic plasticity, and a growth experiment under common conditions (a common garden experiment) is required to visualize and quantify genetic-based phenotypic difference between populations (Savolainen *et al.* 2013). Local adaptation refers to the population differentiation that is attributable to adaptation to local biotic and abiotic environments. To confirm whether the observed population differentiation is the result of local adaptation, the presence of home-site advantage needs to be established by performing a reciprocal transplant experiment; plants from a particular local population have a higher fitness than plants from another population in the original habitat (Kawecki and Ebert 2004).

There is widespread evidence of local adaptation in plants, and there are many variations in the combination of phenotypic traits and environmental factors (Kawecki and Ebert 2004; Savolainen *et al.* 2013). However, in many cases, the underlying genetic mechanisms of local adaptation are unknown. Recently, using natural populations and natural accessions of model species for molecular biology and genetics, such as *Arabidopsis thaliana*, researchers have applied advanced techniques to dissect adaptation genetically (Savolainen *et al.* 2013; Weigel and Nordborg 2015). Genome-wide association studies (GWAS) have identified the genes involved in adaptation to biotic and abiotic environments in *A. thaliana* (reviewed in Bamba *et al.* 2019). For example, genome-wide single-nucleotide polymorphisms (SNPs) have been investigated for associations with the environmental conditions of the source sites (Hancock *et al.* 2011) and with fitness measures in a common garden experiment (Fournier-Level *et al.* 2011). The patterns of local adaptation in *A. thaliana* allowed one to interpret co-variation of multiple traits as a set of life history strategies (Takou *et al.* 2019), such as competitor, stress-tolerator and ruderal (CSR) strategies (Grime *et al.* 1988). Another advanced tool that has recently been used in ecological studies is whole-genome measures of gene expression (transcriptome) analysis with microarray and RNA-Seq (Richards *et al.* 2012; Alvarez *et al.* 2015). Variation in gene expression can be treated as phenotypes that have been analysed in conventional studies of population differentiation and local adaptation. Heritable variation in gene expression can be a target of natural selection, and differentiation in gene expression between populations can be shaped by both adaptive and non-adaptive processes (Oleksiak *et al.* 2002). Therefore, detection of the heritable structures of gene expression variation within and between populations will provide a clue to the underlying genetic mechanisms of local adaptation (Gould *et al.* 2018).

The combination of target traits and environmental factors involved in local adaptation are specific to life history and the habitat of the target species. Therefore, species in the genus *Arabidopsis*, and other closely related species, widen the opportunities for studying diverse traits under selection (Clauss and Koch 2006). The entire genus is now becoming a model system, which allows us to elucidate adaptation with unique combinations of traits and environments, which cannot be studied in *A. thaliana* (Hohmann *et al.* 2014; Yant and Bomblies 2017; Koch 2019). There have been several studies in which *Arabidopsis* relatives were used as a model system to study population differentiation and local adaptation (Turner *et al.* 2010; Leinonen *et al.* 2013). The life history of *A. thaliana* as an annual plant is characterized by a short lifespan, self-compatible flowers and numerous small seeds, and these characteristics are advantageous in a laboratory model plant. Perennial life history, however, will elucidate the adaptation in some important aspects of plant life history, such as outcrossing breeding systems, plant–pollinator interactions, allocation to reproductive and vegetative growth and clonal propagation. Furthermore, annual plants are rare in some habitats, often those with low productivity, e.g. alpine, high-latitude and forest floor habitats. Therefore, adaptation to these habitats requires a study system involving perennial plants.

In this review, we introduced *A. halleri* (L.) O'Kane & Al-Shehbaz as a perennial model system to study population differentiation and local adaptation, amongst which adaptation to altitudinal and edaphic environmental gradients has extensively been studied. This species is characterized by extensive clonal propagation through aerial rosette formation and an outcrossing breeding system by insect pollination. The plant bears leaves all year round, and vegetative parts are exposed to diverse biotic and abiotic stresses. The combination of clonal reproduction and year-round foliage allowed us to repeatedly sample leaves from the same individuals under natural conditions, and has enhanced repeated transcriptome analyses under natural conditions using RNA-Seq. We will highlight a series of RNA-Seq studies using *A. halleri*, and discuss the future applications of transcriptome analysis to studies of population differentiation and local adaptation.

## Arabidopsis halleri

**Figure 1.**
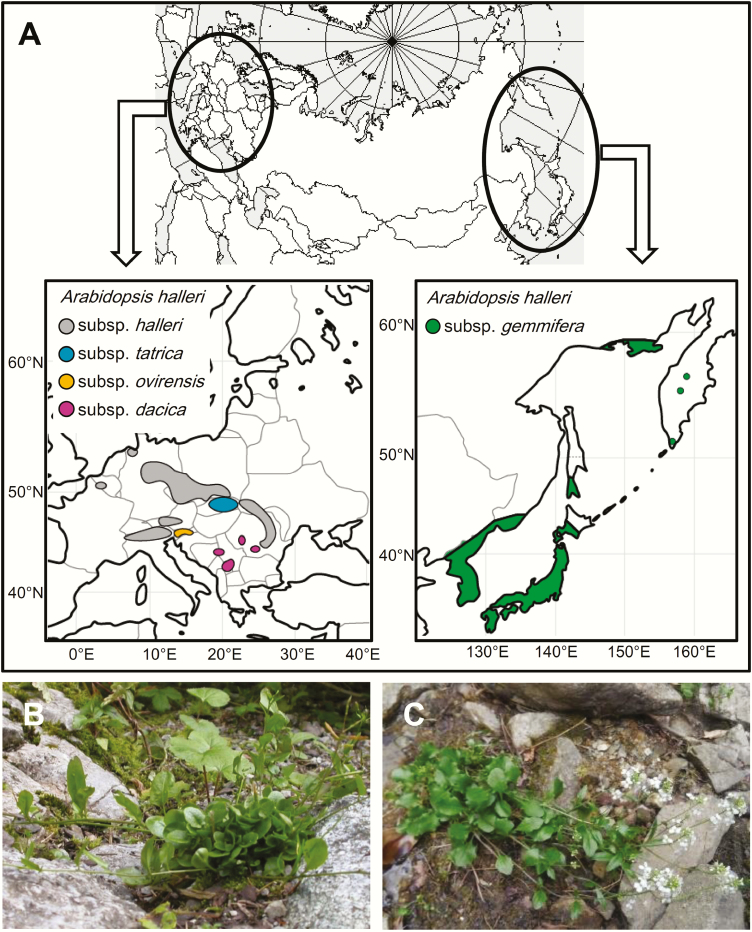
Disjunct distributions of five subspecies of *Arabidopsis halleri* (A). Photographs of *A. halleri* subsp. *tatrica* in Belianske Tatry, Prešovský kraj, Slovakia (B), and *A. halleri* subsp. *gemmifera* in Monzen, Taka-cho, Hyogo Prefecture, Japan (C).

*Arabidopsis halleri* is found in Northern Hemisphere, showing clear disjunction into two part of Eurasia, i.e. East Asia and Europe (Fig. 1A). Four subspecies have been recognized in Europe; *A. halleri* subsp. *halleri*, *A. halleri* subsp. *tatrica* (Pawł.) Kolník (Fig. 1B), *A. halleri* subsp. *ovirensis* (Wulfen) O'Kane & Al-Shehbaz, *A. halleri* subsp. *dacica* (Heuff.) Kolník (Kolník and Marhold 2006; Hohmann *et al.* 2014; Šrámková-Fuxová *et al.* 2017). The eastern distribution is represented by a single subspecies, *A. halleri* subsp. *gemmifera* (Matsum.) O'Kane & Al-Shehbaz (Fig. 1C), which occurs in Japan, Korea, north-eastern China and the Russian Far East, including Sakhalin and Kamchatka (Kudoh *et al.* 2018). All subspecies of *A. halleri* have been reported to be diploid with a basic chromosome number = 8 (2x = 2N = 16; Al-Shehbaz and O’Kane 2002; Kolník and Marhold 2006). The genome size is estimated to be 250 Mb (Briskine *et al.* 2017). The genome assembly has been published and updated (Akama *et al.* 2014; Briskine *et al.* 2017), and 32 553 protein-coding genes were estimated, which is similar to the 32 670 genes of *A. lyrata* (L.) O'Kane & Al-Shehbaz (Hu *et al.* 2011) and >28 775 genes of *A. thaliana* (The Arabidopsis Genome Initiative 2000; Briskine *et al.* 2017). The divergence time from *A. thaliana* has been estimated as 5–18 million years (Koch *et al.* 2000; Ossowski *et al.* 2010), and 0.34–2.5 million years with the more closely related species *A. lyrata* (Castric *et al.* 2008; Roux *et al.* 2011).

## Life History

Comparisons of life history traits between *A. halleri*, *A. lyrata*, *A. arenosa* and *A. thaliana* highlight characteristics of each species (Table 1). Although *A. halleri* is mostly known for its heavy metal hyperaccumulation (Verbruggen *et al.* 2009; Krämer 2010; Stein *et al.* 2017), the species exhibits specific characteristics in its life history different from *A. thaliana* and other species in the genus (Table 1). *Arabidopsis halleri*, *A. lyrata* and *A. arenosa* are all perennial, but *A. halleri* is characterized by clonal reproduction via aerial rosette formation (Table 1). *Arabidopsis lyrata* has been used as a model system to study self-incompatibility (e.g. Kusaba *et al.* 2001; Schierup *et al*. 2001, 2008). *Arabidopsis arenosa* is often used in the study of hybrid incompatibility, because the cross between *A. arenosa* and *A. thaliana* results in viable but infertile F_1_ hybrids (e.g. Burkart-Waco *et al.* 2012), and the one tetraploid species in the genus, *A. suecica*, has shown to be an allotetraploid between *A. arenosa* and *A. thaliana* (O’Kane *et al.* 1996). The interspecies comparison of phenotypes, genomes and transcriptome data provides opportunities to elucidate underlying genetic mechanisms of adaptive traits (Clauss and Koch 2006; Baduel *et al.* 2016; Nikolov *et al.* 2019).

**Table 1.** Comparisons of *Arabidopsis halleri* and related species, *A. lyrata*, *A. arenosa* and *A. thaliana*, in chromosome number, ploidy level, genome size, number of genes, distribution and reproductive and vegetative life histories. NA indicates data not available.

	*Arabidopsis halleri*	*A. lyrata*	*A. arenosa*	*A. thaliana*
Chromosome number	2n = 16	2n = 16	2n = 16 2n = 32	2n = 10
Ploidy level	2x	2x	2x, 4x	2x
Genome size	250 Mbp	230 Mbp	NA	125 Mbp
Number of genes	32 553	32 670	NA	28 775
Original distribution	Europe, East Asia, Far East Russia	Northern Europe, Arctic and Far East Russia, Alaska, Canada	Central Europe	Europe, Central Asia, North Africa
Life cycle	Perennial	Perennial	Perennial, occasionally annual	Annual
Vegetative phenology	Evergreen	Evergreen	Evergreen, winter-green	Winter-green, summer-green
Reproductive phenology	Spring	Spring	Spring	Spring-summer
Breeding system	Obligatory outbreeding	Outbreeding	Obligatory outbreeding	Predominantly inbreeding
Flower size	Petal length = 4–6.5 mm	Petal length = 3–8 mm	Petal length = 5–8 mm	Petal length = 2–3.5 mm
Self-compatibility	Incompatible	Incompatible	Compatible, incompatible	Compatible
Clonal propagation	Formation of aerial rosettes at shoot apical and lateral meristems	Rosettes formation by tillering	Rosettes formation by tillering	None

### Perennial life cycle

The perennial and evergreen life history of *A. halleri* contrasts with the annual life history of *A. thaliana.* Leaves can be produced all year round even in low temperatures during winter (Kudoh *et al.* 2018). Exposure to direct sunlight in midday during winter increases leaf temperatures to the level required for photosynthesis, which is higher than air temperature (H. Kudoh *et al.*, unpubl. data). Recent transcriptome analyses revealed that >80 % of leaf-expressed genes are actively transcribed constantly throughout the year, and furthermore, genes associated with the avoidance of photoinhibition are upregulated during winter (Nagano *et al.* 2019), probably to protect photosynthetic apparatus under low temperature and high light conditions. The timing of the termination of flowering is a phenological trait specific to perennials (Aikawa *et al.* 2010; Satake *et al.* 2013; Nagahama *et al.* 2018), and allocation to reproduction and vegetative growth can be the adaptive traits for study using perennial systems. The perennial and evergreen habits benefit the study of diverse stress under natural conditions, because we can collect sample tissues at any time in a year, and season-specific stresses, such as heat stress during summer, frost during winter, drought during dry season, flooding during rainy season, and herbivore and pathogen attacks during warm periods (Kudoh 2016). Furthermore, it gives opportunities to make comparisons between years using the same individuals.

### Outcross breeding system

This species has obligate outcrossing flowers (Kudoh *et al.* 2016). The flowers are large (4–6.5 mm petal length) compared with those of *A. thaliana* (2–3.5 mm petal length; Table 1; Al-Shehbaz *et al.* 2006). The flowers are self-incompatible, which is sporophytic self-incompatibility similar to other self-incompatible Brassicaceae plants (Kusaba 2001). It has been reported that polymorphisms at the *S* locus in populations of *A. halleri* are shared with those of self-incompatible populations of *A. lyrata*, indicating that ancestral polymorphisms have been maintained by negative frequency-dependent selection acting at the *S* locus (Roux *et al.* 2013). Because of the self-incompatibility, pollen grains need to be transferred through pollinators, and its flowers are often visited by small insects, such as solitary bees, flower flies and bee flies (Kudoh *et al.* 2016). A study conducted in an *A. halleri* subsp. *halleri* population using microsatellite markers estimated a 98.7 % outcrossing rate, and found high multiple paternity within maternal siblings (Llaurens *et al.* 2008). The results suggested that the self-incompatibility is a mechanism to maintain genetic diversity within populations.

Flowering occurs in early spring (Kudoh *et al.* 2018), although the actual timing of flowering in a year should vary depending on the habitat temperature regime (Satake *et al.* 2013). The breeding system of *A. halleri* provides the opportunity to study local adaptation in the traits relating to pollination success. It has been reported that there is a natural selection towards earlier flowering by a flower-feeding leaf beetle in a population of *A. halleri* subsp. *gemmifera* in central Japan (Kawagoe and Kudoh 2010). The *A. halleri* population is highly polymorphic because of the obligate outcrossing enforced by the self-incompatibility of the flowers (Van Rossum *et al.* 2004; Llaurens *et al.* 2008). We expect that the level of inbreeding depression should be high, but there are no measurements available due to the technical difficulties in producing offspring through inbreeding. Self-incompatibility implies some disadvantages to produce a certain set of materials, such as recombinant inbred lines (RIL) and near-isogenic lines (NIL). When the *A. halleri* genome was determined, selfing for five generations was conducted by bud pollinations (Briskine *et al.* 2017), but a more efficient procedure to produce selfing progeny is required to produce RIL and NIL. A self-compatible variant, if it is discovered, will widen the opportunities to develop these materials.

### Clonal propagation via aerial rosette formation

**Figure 2.**
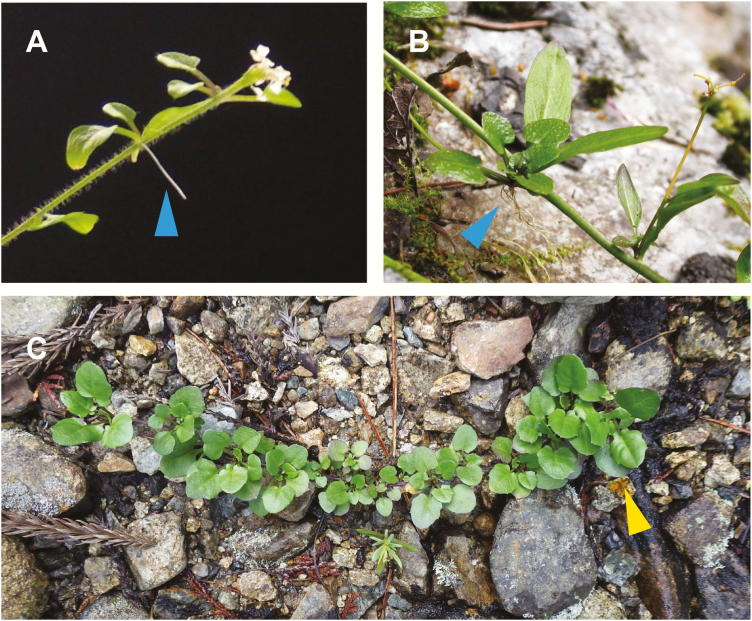
Aerial rosettes formed from lateral meristems along with flowering stems (A and B), and established clonal rosettes (C). Blue triangles indicate roots from aerial rosettes. An orange triangle indicates the position of the original plant. *Arabidopsis halleri* subsp. *gemmifera* in Omoide-gawa, Taka-cho, Hyogo Prefecture, Japan (A and C); *A. halleri* subsp. *tatrica* in Belianske Tatry, Prešovský kraj, Slovakia (B).

Besides sexual reproduction, *A. halleri* plants propagate asexually by forming clonal rosettes (Kudoh *et al.* 2018). The aerial rosettes are formed from lateral meristems, along with the flowering stems at the end of flowering season, for all subspecies (Fig. 2A and B). Even apical reproductive meristems, that have produced flowers, return to vegetative meristems at the end of flowering season (Aikawa *et al.* 2010; Kudoh *et al.* 2018), and the phenomenon is known as inflorescence reversion (Tooke *et al.* 2005). Roots are produced at the bases of aerial rosettes when they are in the air (Fig. 2A and B). Eventually, flowering stems lay down and some of the aerial rosettes successfully establish by penetrating roots into the soil (Fig. 2C). This conspicuous mode of clonal propagation is specific to *A. halleri* and is in contrast with *A. lyrata* and *A. arenosa* (Table 1).

The distance between clonal rosettes is restricted, and the size of the patches, consisting of genetically identical multiple rosettes, is usually <30 cm in diameter, occasionally 30 cm to 1 m and rarely 1–2 m, as found in a population of *A. halleri* subsp. *gemmifera* (M. N. Honjo and H. Kudoh, unpubl. data). Therefore, a single population usually contains several hundred to several thousand scattered and intermingled clonal patches, each originating from a single seed. Therefore, both clonal propagation and seed production are critical for the maintenance of *A. halleri* populations. In a population of *A. halleri* subsp. *halleri* in France, a study using microsatellite markers reported that clonal spread occurs only at short distances <1 m, and high clonal diversity (*D*_G_ > 0.9) is maintained by sexual reproduction (Van Rossum *et al.* 2004). Clonal population structures can be studied in further detail by analysing genome-wide SNPs and applying new techniques, such as RAD-Seq (Miller *et al.* 2007) and Mig-Seq (Suyama and Matsuki 2015), to estimate the kinship between clones and level of somatic mutation between clonal individuals. When conducting growth experiments, clonal propagation offers an easy way to get plants for experiments, especially taking into account the high parental diversity found in some of the *A. halleri* populations (Llaurens *et al.* 2008).

The habitat of *A. halleri* is generally characterized by a high frequency of natural disturbances, and therefore the turnover rate of existing rosettes is high (H. Kudoh, unpubl. data). The formation of clonal rosettes assures the continuation of clonal lineages by spreading the risk of mortality across clonally replicated rosettes (i.e. ramets of the same genet). As well as risk spreading, multiple rosettes may be physiologically integrated through connections between them, to cope with the spatial heterogeneity of environments (Gruntman *et al.* 2017). We expect that clonal propagation through aerial rosettes has evolved in response to habitat disturbance or habitat productivity. It has been reported that clonal spread is more extensive in less heavy metal-contaminated locations than in highly contaminated locations in a population of *A. halleri* subsp. *halleri* (Van Rossum *et al.* 2004).

## Studies on Population Differentiation and Local Adaptation

Here, we review previous studies on population differentiation and local adaptation, frequently studied along two major environmental gradients. The first one is altitudinal adaptation, and it has been studied extensively in two regions, i.e. at Mt. Ibuki, Japan for *A. halleri* subsp. *gemmifera* and in the European Alps for *A. halleri* subsp. *halleri*. Although both studies focus on a population that occurs in an altitudinal environmental cline, the background settings largely differ. The second gradient is heavy metal concentration in the soil. Population differentiation has been studied between habitats with metalliferous and non-metalliferous soils. Because *A. halleri* is a representative plant that shows heavy metal hyperaccumulation, there has been a strong demand to understand its mechanisms for use in phytoremediation of heavy metal-contaminated soils, which has led to the accumulation of information on this aspect.

### Altitudinal adaptation

Plant populations distributed along altitudinal gradients experience a wide range of abiotic and biotic environmental conditions, depending on altitude and aspect of slopes (Körner 2007). Plants are likely exposed to stressful environments, such as freezing temperature, drought and ultraviolet-B (UV-B), with subtle topographical variation substantially influencing plant fates. Such steep ecological and environmental gradients are likely to cause strong selection and lead to population differentiation and local adaptation. Because altitudinal gradients provide contrasting habitats within a short geographic distance, gene flow at this scale might be more effective at countering the demographic effects of populations than at larger scales. Altitudinal gradient, therefore, provides an opportunity to identify traits under selection (Gonzalo-Turpin and Hazard 2009). In the application of genome scans, outlier loci will probably represent adaptive genetic variation rather than statistical outliers, resulting from historical processes affecting the neutral genetic variation. Thus, *A. halleri* populations from contrasting habitats along altitudinal gradients offer excellent opportunities to study genetic differentiation and local adaptation.

**Figure 3.**
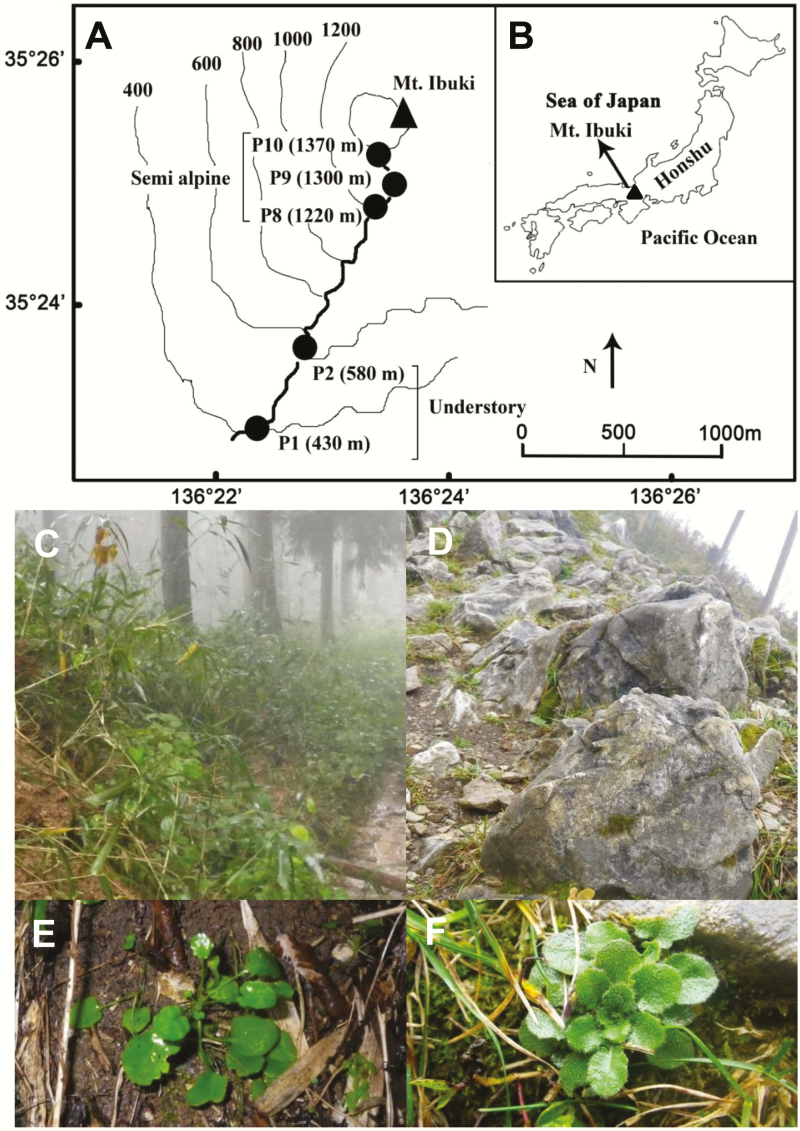
Natural populations of *Arabidopsis halleri* subsp. *gemmifera* occurring along the altitudinal gradient at Mt. Ibuki, Shiga Prefecture, Japan (A–F). A map showing locations of natural populations in low-altitude understory habitat and in high-altitude semi-alpine habitat (A). A map showing the location of Mt. Ibuki (B). Photographs representing environments of understory (C) and semi-alpine habitats (D). Rosettes of *A. halleri* under rainy conditions for understory (E) and semi-alpine habitats (F). In (A), a hiking route is shown by a thick line, and P1, P2, P8, P9 and P10 represent pitches 1, 2, 8, 9 and 10 along the route. Altitudes are in parentheses.

Mt. Ibuki, where *A. halleri* subsp. *gemmifera* inhabits, is located in central Japan (altitude 1377 m at the highest peak; Fig. 3A). Honshu Island, the main island of Japan, has an elongated shape but is characterized by contrasting winter weather conditions between the Sea of Japan and Pacific sides. Prevailing north-west winds from the cold Asian continental region absolve moisture when they cross the Sea of Japan and bring heavy snow to the Sea of Japan side of Honshu. Mt. Ibuki is a freestanding peak that is located on the narrowest part of Honshu Island and on the border of the Sea of Japan and Pacific weather areas (Fig. 3B). This specific location characterizes the mountain with a combination of cold wind and snow near the top and relatively warm and mild winter weather near the base.

The contrasting habitats of *A. halleri* subsp. *gemmifera* on Mt. Ibuki are classified into two types: one is the edges and understory of the *Cryptomeria japonica* forests near the base of mountain (referred to as understory hereafter; Fig. 3C), and the other is the semi-alpine open habitat near the top (referred as to semi-alpine hereafter; Fig. 3D). In the understory of Mt. Ibuki, *A. halleri* plants are glabrous (Fig. 3E). Plants in the semi-alpine habitat of Mt. Ibuki have a distinctive increased density of trichomes on the leaves (Fig. 3F) compared to typical *A. halleri* plants (glabrous – sparsely hairy), and are sometimes treated as a variety of *A. halleri* subsp. *gemmifera* [originally described as *Arabis gemmifera* (Matsum.) Makino var. *alpicola* H. Hara (Hara 1936; Ihara 1976)]. Al-Shehbaz and O’Kane (2002) treated the name as a synonym of *Arabidopsis halleri* subsp. *gemmifera*, and Yonekura (2011) treated it as *A. halleri* subsp. *gemmifera* f. *alpicola* (H. Hara) Yonek.

It has been reported that the level of genome-wide genetic differentiation between the low- and high-altitude populations on Mt. Ibuki was low (*F*_st_ = 0.017) in the amplified fragment length polymorphism (AFLP) analysis (Ikeda *et al.* 2010). The analysis using 19 microsatellite loci for 41 populations from a wide-ranging area also resulted in a close genetic similarity for the populations on Mt. Ibuki and the surrounding area (Sato and Kudoh 2014). The altitudinal gradient on Mt. Ibuki, therefore, has often been selected as a model system to study population differentiation and local adaptation.

Ultraviolet-B (280–315 nm in wave length) radiation is a critical abiotic factor increasing ecological stress in plant populations at high elevations. Although the peak of Mt. Ibuki is not high, the habitat of *A. halleri* in the semi-alpine area is exposed to much more direct sunlight, compared with the understory habitat in lower altitudes. Wang *et al.* (2016) conducted a growth chamber experiment and found an altitudinal differentiation in the response to UV-B damage. Plants from seeds collected at 380, 760 and 1300 m in elevation on Mt. Ibuki were grown with and without supplemental UV-B. They used the level of cyclobutane pyrimidine dimer (CPD) as an indicator of the level of DNA damage. They applied a UV-B treatment to plants grown for 22 days after seedling transplantation. Thereafter, CDP levels were measured in leaves after 10 and 40 days of the treatment (early and late stages, respectively). At the early stage, plants from the lowest altitude exhibited a higher CPD level and greater inhibition in biomass production, indicating that low-altitude plants were more sensitive to increased UV-B than high-altitude plants. In contrast, at a later stage, CPD for plants from the lowest altitude decreased, and CPD level and growth inhibition became similar across plants that originated from different altitudes. The response to UV stress was concluded to be constitutive in highland ecotypes, but more inducible in lowland ecotypes (Wang *et al.* 2016). The results suggested the population differentiation occurred under the constraint of a trade-off between growth and UV tolerance (Wang *et al.* 2016).

Leaf wettability is an indicator of the water affinity/repellency of the leaf surface, and measured by the contact angle of a droplet when a designated amount of water is applied to the leaf surface. When leaf wettability is high water spreads on the leaf surface, while water forms a droplet on the non-wettable leaves, and the leaf surface is kept dry in the rain and fog. Aryal *et al.* (2018) found cauline-leaf-specific genetic differentiation in leaf wettability between high- and low-altitudinal plants on Mt. Ibuki. *Arabidopsis halleri* produces two types of leaves, i.e. rosette and cauline leaves. Rosette leaves are produced all year round and the adaxial (upper) surface is exposed. Cauline leaves are produced prior to flower-bud formation and later become leaves on the flowering stems. During early spring, cauline leaves wrap young flower buds, and the abaxial surfaces are exposed. In the results of a field survey and a common garden experiment using a growth chamber, cauline leaves of semi-alpine plants, especially on abaxial surfaces, turned out to be non-wettable, whereas cauline leaves of low-altitudinal understory plants were wettable (Aryal *et al.* 2018). There were no altitudinal differences in the leaf wettability of rosette leaves. As a candidate gene determining the leaf wettability pattern, the gene expression of a key gene for alkane biosynthesis, *AhgCER1*, was examined, and the gene was highly upregulated in cauline leaves of high-altitudinal plants (Aryal *et al.* 2018). Alkane is known to be a highly hydrophobic molecule that is accumulated in the epidermal wax of *Arabidopsis* leaves (Jetter and Kunst 2008). Dry surfaces of cauline leaves are hypothesized to protect flower buds from morning frost at the higher altitudes of Mt. Ibuki in early spring (Aryal *et al.* 2018).

Although local adaptation has not yet been rigorously tested, an edaphic factor may be an important cause of population differentiation. High-altitudinal habitats are characterized by calcareous soil, whereas habitats with non-calcareous soil exist at lower altitudes. Wang *et al.* (2019) reported altitudinal population differentiation of *A. halleri* at Mt. Ibuki in response to soil nitrogen availability. It has been previously reported that naturally growing plants at high altitude on Mt. Ibuki accumulated less zinc than plants growing in non-calcareous habitats at lower altitudes (Kosugi *et al.* 2015).

Taking advantage of the small genome size, and being closely related to *A. thaliana*, genome scans have been applied to identify SNPs that show differentiation associated with habitat environments (Kubota *et al.* 2015). Genome scans have been used to identify the footprints of selection across the genome to pinpoint the loci under selection. Kubota *et al.* (2015) applied the method to plants from different altitudes of Mt. Ibuki, as well as another mountain nearby with a similar altitude at the highest peak, Mt. Fujiwara. They conducted individual-base re-sequencing of 56 plants and analysed 527 225 SNPs. For genome-wide SNPs, *G*_st_ = 0.043–0.048, indicating low population differentiation at the whole genome level. They successfully identified candidate genes for altitudinal differentiation, and their functional annotations involved those related to altitudinal adaptation, such as cold and freezing tolerance. Moreover, two genes involved in the convergent evolution on the two mountains were identified: homologs of *GLUCAN SYNTHASE-LIKE 8* (*GSL8*) and *PBA1*. The former is related to growth form and the latter encodes proteasome subunit beta, which is related to diverse types of stresses (Kubota *et al.* 2015). Quantifying the allelic effects of these genes on fitness by common garden and reciprocal transplant experiments will be required to understand how selection has acted on this particular genome region.

Altitudinal variation in local herbivore pressure is predicted to be a selection agent that causes population differentiation in traits related to plant defence (Løe *et al.* 2007; Halbritter *et al.* 2018). This aspect has been studied using populations of *A. halleri* subsp. *halleri* from wide-ranging altitudes of the European Alps (Buckley *et al.* 2019). They found a lower degree of herbivore damage at higher altitudes in the field populations, occurring at different altitudes ranging from 300 to 2300 m. In a common garden experiment, plants showed a cline of population differentiation in association with indole glucosinolates, which imparted constitutive chemical defence. They revealed that relative quantities of indole glucosinolates significantly increased with elevation, and were negatively correlated with herbivore damage in the field. In oviposition preference assays, females of a white butterfly, *Pieris brassicae*, laid fewer eggs on plants obtained from high-elevation populations (Buckley *et al.* 2019). These results suggest that *A. halleri* plants at high altitude are genetically well defended against herbivory.

A genome scan study has been conducted using five populations of *A. halleri* in the Alps, ranging from 790 to 2308 m in altitude, to characterize genetic variation and identify genes associated with climatic variation (Fischer *et al.* 2013). They pooled population samples (20 individual plants per population) for whole-genome sequencing (Pool-Seq) and estimated allele frequency of populations at SNP loci. Highly differentiated genomic regions and SNPs were identified from two million SNPs using *F*_st_-based analyses (Fischer *et al.* 2013). They found that the most strongly differentiated genomic regions in *A. halleri* were typically small and widely dispersed across the genome. By applying partial Mantel tests, 175 genes were identified to be highly associated with one or more of the five topo-climatic factors, i.e. temperature, precipitation, solar radiation, angle of slope and water balance (Fischer *et al.* 2013). Allele frequencies of two genes [homologs of *P-GLYCOPROTEIN 1* (*PGP1*) and *GLUTAMATE RECEPTOR 3.6* (*GLR3.6*)] were strongly associated with solar radiation, and those of two other genes [homologs of *LOW OSMOTIC STRESS 5* (*LOS5*) and *GLUTATHIONE PEROXIDASE 3* (*GPX3*)] were strongly associated with water balance. Furthermore, their gene annotations were related to responses to the associated environmental factors (Fischer *et al.* 2013). Later, larger scale analyses with 444 plants from 18 populations of *A. halleri* (ranging from 309 to 2305 m) were reported (Rellstab *et al.* 2017). Using two data sets, they narrowed down environmentally associated genes to 11 loci, and concluded that these genes play a significant role in large-scale adaptation to the abiotic environment. One of the examples was a homolog of *SYNC1*, which was related to seed dormancy, and two SNPs in the locus showed an association with precipitation (Rellstab *et al.* 2017).

### Adaptation to metalliferous soils

Metalliferous habitats exert strong selection pressure on plants by the accumulation of heavy metals at toxic concentrations in plant tissues, and interferences with the plant metabolism (Antonovics *et al.* 1971; Macnair 1987). Under such circumstances, one expects to observe population differentiation and local adaptation in heavy metal tolerance between metalliferous and non-metalliferous (referred to as M and NM hereafter) sites. The rapid evolution of heavy metal tolerance in response to recent exposure to anthropogenic metal polluted areas is a classic example of local adaptation (Antonovics *et al.* 1971; Baker and Brooks 1989; Pollard *et al.* 2002). In these cases, species originally sensitive to heavy metals evolve, via local adaptation in populations exposed to high concentrations of heavy metals, to be resistant or tolerant to this toxicity (Antonovics *et al.* 1971; Macnair 1987).

Metal hyperaccumulation is the ability to allocate extraordinarily large amounts of metals to shoots, without showing any toxicity symptoms (Antonovics *et al.* 1971). Hyperaccumulation is widely observed in plants; however, the levels of quantitative variation among populations and between individuals within populations can vary considerably (Gonneau *et al.* 2014; Stein *et al.* 2017). This is true for *A. halleri*, for which heavy metal hyperaccumulation and heavy metal tolerance seem to be the rule, irrespective of the provenance of populations (i.e. M or NM habitats) (Meyer *et al.* 2015; Stein *et al*. 2017). Therefore, evolution of heavy metal tolerance in *A. halleri* is likely to have a long history that involves multiple complex mechanisms (Pauwels *et al.* 2006). The population differentiation of metal hyperaccumulation and local adaptation of heavy metal tolerance in *A. halleri* have been reported as a form of enhanced tolerance; plants from M populations are more tolerant than those from NM populations to high Zn concentrations (Pauwels *et al.* 2006; Meyer *et al.* 2010) and Cd (Meyer *et al.* 2015) in population averages. Generally, a large standing variation seems to be maintained both for M and NM populations (Bert *et al*. 2000, 2002; Stein *et al.* 2017).

To evaluate Zn tolerance of scattered European populations of *A. halleri* in M and NM sites, Pauwels *et al.* (2006) measured survival rates experimentally under conditions of Zn concentration ranging from 1 to 2000 µM. They found Zn tolerance in all examined *A. halleri* populations. Plants from the M populations were the most tolerant; however, the ranges of variation in tolerance overlapped between NM and M populations. Moreover, relatively high levels of tolerance were detected in some NM populations. They considered that highly tolerant plants have been maintained in some populations without encountering heavy metal exposure, and they termed the phenomenon constitutive tolerance. Later phylogenetic analyses revealed the existence of two genetic groups in Europe that originated from geographic isolation during the glacial period, and the enhancement of heavy metal tolerance in anthrophonic contaminated sites occurred independently in each genetic group (Pauwels *et al.* 2012). We also should consider current gene flow between M and NM populations, which may make a contribution to the existence of enhanced tolerance in NM population.

Meyer *et al.* (2010) examined genetic variation in Zn tolerance between seven M and five NM populations in Poland and Slovakia. To test the hypothesis that divergent selection has shaped this polymorphism, the morphological and physiological traits of shoots and roots were measured to quantify the response of *A. halleri* to Zn exposure. On average, tolerance levels measured were higher in plants from the M populations than those of the NM populations. Phenotypic variability was high and mostly accounted for the differences between individuals within populations. Genetic differentiation (*Q*_ST_) for the photosystem II yield of leaves (a measure of photosynthetic efficiency) was large in contrast to the small differentiation (*F*_ST_) estimated from 10 microsatellite loci and thus was probably shaped by divergent selection. They discussed how Zn tolerance is increased in M populations by selection, which acts on the standing genetic variation within an ancestral NM population.

Babst-Kostecka *et al.* (2018) investigated the population genetic structure of eight M and six NM populations of *A. halleri* in Poland using 10 microsatellite loci. Different geographical groups, rather than M and NM separation, were identified. Genetic variation in Zn hyperaccumulation was detected in all the investigated populations, suggesting that Zn hyperaccumulation can respond to selection. Moreover, they suggested that the current distribution of *A. halleri* in southern Poland could be a result of habitat fragmentation caused by climatic shifts after the last glacial period, rather than due to the recent colonization of industrially polluted sites. In addition, some lowland NM populations may have been derived from M populations. They concluded that Zn hyperaccumulation evolved both ways, towards higher levels at NM sites and lower levels at M sites.

In most of the reported studies, Zn accumulation level in shoots was higher in plants from NM populations than that in plants from M populations under the common experimental conditions (Bert *et al.* 2000; Stein *et al.* 2017; Babst-Kostecka *et al.* 2018). A similar pattern has been reported for Cd accumulation (Stein *et al.* 2017). Therefore, the function of Zn and Cd hyperaccumulation of *A. halleri* in NM habitats remains an intriguing question. Multiple hypotheses regarding the function of the metal hyperaccumulation have been discussed (Boyd and Martens 1992). Initially, because absolute metallophytes often show hyperaccumulation, it was considered to be a part of physiological tolerance against the high level of metals in soil (Baker 1981). However, it remained unsolved how metal hyperaccumulation enhances plant fitness. More recently, the ‘elemental defence hypothesis’, in which high metal concentration in plant tissues acts as a defence against certain herbivores or pathogens, has been tested in many studies (Boyd 2004, 2007; Fones *et al.* 2010; Rascio and Navari-Izzo 2011; Kazemi-Dinan *et al.* 2014; Stolpe *et al.* 2017). Assuming that similar levels of metal concentration are required for defence in M and NM populations, one may expect that increased hyperaccumulation ability for plants in the latter populations.

As we have seen in the studies of altitudinal adaptation on Mt. Ibuki and in the Alps, a genome scan by whole-genome re-sequencing of population pools (Pool-Seq) was conducted to identify SNPs that associate with heavy metal concentration in natural habitats (Sailer *et al.* 2018). They used two M and two NM populations (*N* = 119 plants) and identified 57 SNPs in 19 genes significantly associated with soil adaptation. These genes included those related to transmembrane transport and responses to stress. One example is *METAL TOLERANCE PROTEIN A2* (*MTPA2*). Therefore, both translocation of heavy metals and detoxification processes are likely to be important in the adaptation to M and NM soils.

Quantitative trait locus (QTL) mapping, transcriptome studies and functional analyses have resulted in critical progression of our understanding of the molecular mechanisms underlying metal tolerance and accumulation in *A. halleri* (reviewed by Verbruggen *et al.* 2009; Krämer 2010). For Zn tolerance and accumulation in *A. halleri*, constitutive high expression levels of genes involved in root uptake (members of the ZIP gene family, a metal transporter family first identified in plants), root-to-shoot translocation [*HEAVY METAL ATPAse 4* (*HMA4*) and *NICOTIANAMINE SYNTHASE 2* (*NAS2*)] and vacuolar sequestration [*METAL TOLERANCE PROTEIN 1* (*MTP1*)] play a central role. For Cd tolerance, *HMA4* also has an important function in root-to-shoot metal translocation and metal distribution in shoots (Hanikenne *et al*. 2008, 2013). Furthermore, *HMA4* has been reported to be triplicated in *A. halleri* (Hanikenne *et al*. 2008, 2013). The knowledge on the actual genes involved in hyperaccumulation and tolerance could provide us with a great advantage in studying population differentiation and local adaptation in *A. halleri*.

## RNA-Seq – Widening Phenotype Space

Next-generation sequencing (NGS) is being used increasingly for the study of population differentiation and local adaptation. In the above examples, as in the method to analyse population differentiation using whole genome SNPs, genome scans using individual-based re-sequencing and the re-sequencing of population pools (Pool-Seq) have been applied in the study of *A. halleri*. Here, we introduce RNA-Seq methods that can be used in future studies of population differentiation and local adaptation of *A. halleri*.

### High-throughput RNA-Seq

Transcriptome analyses using RNA-Seq have become a standard method in molecular biology to compare multiple genotypes (e.g. wild type vs. mutant) and to evaluate environmental responses. Thus, application of RNA-Seq in the study of population differentiation and local adaptation is expected. The quantitative measures of gene expression can be treated as conventional measures of phenotypic traits for samples from field populations, common garden experiments and reciprocal transplant experiments (Gould *et al.* 2018). Therefore, the key innovation is whether it is applicable for multiple samples. Development of high-throughput and cost-effective methods for RNA-Seq library preparation, and the application of these methods to target species is required. Several methods have been already developed (Wang *et al.* 2011; Townsley *et al.* 2015).

Application of high-throughput RNA-Seq has been done previously in a natural population of *A. halleri* subsp. *gemmifera* in central Japan (Kudoh *et al.* 2018). As a molecular phenology study in the population (Kudoh 2016), leaf transcriptome data from six *A. halleri* plants were obtained weekly for 2 years, and bi-hourly for 2 days on spring and autumn equinoxes, and summer and winter solstices (Nagano *et al.* 2019). It has been reported that 16.7 and 41.8 % of the leaf-expressed genes (2879 and 7185 out of 17 205, respectively) show seasonal and diurnal oscillations (Nagano *et al.* 2019). Although the study did not aim to analyse population differentiation and local adaptation, it reveals an important property of gene expression when we use it as a phenotypic trait; i.e. the sampling time in a day needs to be strictly controlled, because many genes exhibit diurnal changes in their expression levels. The tissue, time and environment specificity in gene expression will be important to interpret the observed variation in gene expressions between and within populations.

Transcriptome data have three innovative properties that give us advantages in the study of population differentiation and local adaptation; first, a comprehensive phenotype set, second, a linker between the phenotype of ecological traits and underlying genetic basis, and third, a trait set that gives a clue to identifying the causal nucleotide substitution(s). As per the first property, RNA-Seq data include genes that are involved in wide range of morphological and physiological traits. Because of this comprehensiveness, we can explore in which traits populations are differentiated without targeting particular traits in advance. As per the second property, gene expression data are more closely linked to underlying genetic mechanisms than phenotypes of ecological traits. Therefore, the detection of differentially expressed genes (DEGs) between populations allows us to identify which part of the gene regulation network is under selection and/or is responsive to selection. As per the last point, the causal nucleotide substitution(s) may be detected in RNA-Seq sequences when they exist in transcribed regions. Causal substitution(s) might be located in the regulatory region of most upstream DEGs in the regulatory network of targeted phenotypic traits. With the combination of SNP analyses on the genome, it could help elucidate the causal nucleotide substitution(s) responsible for population differentiation and local adaptation.

### Dual RNA-Seq

In RNA-Seq, NGS determines all RNA reads in a prepared library, and therefore not only the RNAs of the target tissues, but also RNAs derived from endogenous organisms, can be subjected to further analyses. Simultaneous analysis of hosts and parasites has been termed as dual RNA-Seq (Westermann *et al.* 2012), and the idea can be applied to any endogenous organisms including pathogens, parasites and symbionts. In the previous examples, transcriptomes of pathogenic endogenous fungi and host plants were analysed simultaneously (Tierney *et al.* 2012; Westermann *et al*. 2012, 2016). The method can be applied to the study of population differentiation and local adaptation for both host and endogenous organisms.

A method to identify all infected plant viruses using RNA-Seq has been developed (Nagano *et al.* 2015). Because this method targets total RNA, except plant rRNA, in contrast to commonly used methods, which target mRNA, a broad range of viruses can be detected. It has been reported that five viruses, *Turnip mosaic virus* (TuMV), *Brassica yellows virus* (BrYV), *Cucumber mosaic virus* (CMV), *Pelagonium zonate spot virus* (PZSV) and *Arabidopsis halleri partitivirus 1* (AhPV1), can infect naturally growing *A. halleri* subsp. *gemmifera* (Kamitani *et al*. 2016, 2017). Endogenous organisms might act as environmental factors that can result in divergent selection between populations. The TuMV infection rates were compared between multiple natural populations, and ranged from 0 to 57 % (Kamitani *et al.* 2019). We still have limited knowledge on the regional variation in virus infection rates and whether this geographic variation is translated into differentiation among *A. halleri* populations.

An original dual RNA-Seq for the simultaneous analysis of host and pathogen transcriptomes (Westermann *et al.* 2012), virus accumulation and host transcriptome can be examined simultaneously in the plant virus–host system (Kamitani *et al.* 2016). Dual RNA-Seq employed for TuMV–*A. halleri* systems has revealed that the level of virus accumulation changes dynamically within the host and virus infection affects host transcriptomes by altering the expression of genes related to defence responses and flavonoid biosynthesis during long-term persistent infections (Honjo *et al.* 2019). When the relationship between the host and endogenous organisms is persistent, we may expect co-evolution between host plants and endogenous organisms. It has been considered that there exists a trade-off between virulence and transmission rates. How such relationships between host and endogenous organisms affect population differentiation and local adaptation is an open question that can be addressed in future using *A. halleri* as a target species.

### Epigenetic modifications

Epigenetic modifications, such as DNA methylation and post-transcribed histone modification, determine chromatin states that alter gene expression, and can play a critical role in the regulation of phenotypes in response to local environments. Some classes of epigenetic modification have been reported to show environmental responses for long periods, sometimes even across generations (Verhoeven *et al.* 2016). As DNA methylation and histone modification can be measured at a single nucleotide and a couple of 100 bp, respectively, measurements for each gene can be utilized to interpret the results of RNA-Seq. It is noteworthy that methods to analyse the epigenome of *A. halleri* under field conditions have become available; analyses on histone modifications using chromatin immunoprecipitation followed by quantitative PCR (ChIP-qPCR) or next-generation sequencing (ChIP-Seq), and DNA methylation using bisulphite sequencing (Nishio *et al.* 2016; Ito *et al.* 2019).

## Conclusions

In conclusion, *A. halleri* provided us with opportunities to study population differentiation and local adaptation, especially along altitudinal gradients and non-metalliferous–metalliferous soil gradients. In the former examples, diverse factors such as temperature, UV-B, frost, soil nutrients and herbivores have been identified as selection agents. In the latter examples, *A. halleri* plants show heavy metal hyperaccumulation irrespective of the origins, and plants from metalliferous habitats exhibited enhanced heavy metal tolerance. Therefore, in either adaptation to high-altitudinal environments or to heavy metal-contaminated soils, alternation in multiple genetic pathways is likely to be involved in the adaptation processes. The results of genome scans also supported this hypothesis, and owing to the close relatedness to *A. thaliana*, most of the candidate genes under selection have been annotated, making it easier to infer their function. By combination with rigorous common garden experiments, growth chamber experiments and reciprocal transplant experiments, gene expression data could provide critical information to elucidate genetic systems underlying observed population differentiation and local adaptation. Because we now have lists of the candidate genes responsible for local adaptation, the effort of connecting expression differences in these genes with fitness outcomes in natural habitats has become critically important.

## Sources of Funding

This study was supported by Japan Science and Technology Agency (JST), CREST no. JPMJCR15O1 and Japan Society for the Promotion of Science (JSPS) Grant-in Aid for Scientific Research (A) no. 19H01001.

## Contributions by the Authors

M.N.H. and H.K. conceived the idea, collected literature and wrote the manuscript.

## Conflict of Interest

None declared.
